# Impressions in Plaster of Paris

**Published:** 1855-01

**Authors:** S. Mallett

**Affiliations:** New Haven


From the Dental Recorder.

### IMPRESSIONS IN PLASTER OF PARIS.

MR. EDITOR:

In compliance with your request, I will give you some practical thoughts on taking Plaster Impressions; and in doing so, I do not expect to say anything that will be *new* to the most experienced and profound of my professional brethren. That which is plain and unvarnished will not, I trust, be unacceptable to a few of your readers, inasmuch as, I suppose, it is the business of the journalist to bring forth not only things which are new, but also to spread before the minds of his numerous readers those things which, although they have not the novelty of *new inventions*, are, nevertheless, interesting and useful to those who do meet with them for the first time.

There are so many at the present day who are just entering the profession, as well as a great number who are already in the practice of dentistry, and have not had the most favorable opportunity to learn the modern improvements in Mechanical Dentistry, and are not familiar with all the best modes of manipulation—that anything practical relating to these subjects, although it may be familiar and common place to the more experienced, will not prove unacceptable to many of your readers. Perhaps the better informed will be conciliatory towards the uninformed, while we say something which is already well-known to many. In order to take good plaster impressions, two or three things are essential. First, there must be a suitable *cup* or *holder* provided to receive and retain the plaster. To secure this, some first take a wax impression in an ordinary holder, and then after it has become suitably hardened, it is trimmed, and enlarged around the rim of the impression to make room for the waste plaster. A very thin mixture of the plaster is then put into the impression, and spread on the surface of the wax where the impression is designed to be made.

The wax impression should not be filled with the plaster. The wax being so near the form of the mouth, it is only necessary to cover the wax with a thin coating of plaster.

A better way than this is, to take a block-tin or britannia holder, formed as near the shape of the parts designed to be copied as possible, and the best holders I have seen for this purpose are some I recently found at the Dental Salesrooms in New York. They are heavy Britannia holders, made on casts of the mouth. To adapt these holders to the purpose of taking impressions in Plaster of Paris, I cut and bend them, (which can be easily done,) into such shape as best suits the case in hand.

They are susceptible of being cut or bent as easily as lead, though this may sometimes destroy the holder for future use.

For difficult cases, after taking an impression, a plate should be struck up of some base metal—it may be brass or copper. This plate is then used as the plaster holder, the edge of the plate being bent outward, somewhat, to make room for the plaster. There should always be a small orifice drilled in the arch of the *holder* of the upper jaw, to prevent the impression adhering to the mouth, by atmospheric pressure, which will prove a serious hindrance in removing the holder, if this precaution is not taken.

The under jaw always presents the most difficulty in securing a perfect impression with the ordinary holder, with the use of plaster; but this may generally be taken with wax, with more ease than that of the upper jaw. The swaged plate, as a holder for plaster, may always be depended upon, when other modes have failed of success.

Where there has been a failure to make a perfect fit, the plate prepared for insertion, may be used as a holder. If the proper course is pursued, in taking the impression, the under jaw requires much less labor in adapting a plate to it than the upper jaw, and there is none the less surety of success.

A suitable holder having been procured, the next important step is to mix the plaster, with water and salt in suitable proportions, and adjust the paste to its place in the mouth, just at the moment it has acquired the proper stiffness. In order to accomplish this, considerable dexterity must be used at the moment the plaster is ready to be introduced into the mouth. Also, it requires some discrimination, (which can be acquired only by practice,) to know when to proceed with this part of the operation. However, if the following course is pursued, there need be no difficulty here, viz: Wet up the plaster to about the consistency of cream, and instead of making it stiff, by adding more plaster, let it stand until it can be heaped for a moment; and now, using care not to overfill the holder, no time should be lost in introducing it to its place in the mouth. The gums and every part of the mouth designed to be impressed, should be brought in contact with the paste,) the inner portion of the holder approaching first,) and bedded in it with one uniform, gentle, but firm pressure, mostly on the central portion of the holder, and in the same steady manner should it be held until the plaster is hard enough to be removed without breaking. Before the plaster has become very stiff, a small crooked instrument, prepared for the purpose, and lying near at hand, should be passed through the orifice in the plate, and piercing through the plaster to the roof of the mouth, being sure, by some sign from the patient, that the instrument has reached thus far. Just before taking out the apparatus, the instrument should again be applied, so as to be sure that the air can be freely admitted between the plaster and the palatal arch. The best impressions cling the closest to the mouth, and after the introduction of air, may be easily removed.

Before taking an impression, the precaution should be observed to place the patient in as upright a position as convenient, and after the holder is introduced, the patient should lean forward, dropping the face downward, to prevent the plaster from running down the throat.

A napkin should be laid over the chest of the patient, and another held under the chin while the operation is being performed, to catch the saliva, or plaster, that might drop. It is advisable, in order not to produce any nervous haste, in the patient, to suggest that when all is ready, you will be obliged to proceed with great promptness, in order to use the plaster before it becomes too hard.

For partial sets, to be held by atmospheric pressure, it is always essential to make a holder by swaging up a plate over a cast of the mouth. A very thin covering of plaster will be enough on the palatal surface of such a holder. In this case, the plaster should not stand long before being applied to the parts of which an impression is to be made, but used immediately after being prepared. Before removing the plate or holder, a small, sharp pointed instrument should be passed around the edges of the plate and necks of the teeth, in order to accurately trim the plaster that it may not break in removing. An atmospheric plate may be fitted for as small a number as two or three, or even one tooth that will answer a good purpose. I have succeeded with cases in this manner, that had been considered impracticable by others.

S. MALLETT.

*New Haven*, *November*, 1854.

